# Venous Thromboembolism in Children with Cancer and Blood Disorders

**DOI:** 10.3389/fped.2017.00012

**Published:** 2017-02-06

**Authors:** Richard H. Ko, Courtney D. Thornburg

**Affiliations:** ^1^Genentech, Inc., South San Francisco, CA, USA; ^2^Hemophilia and Thrombosis Treatment Center, Rady Children’s Hospital San Diego, San Diego, CA, USA

**Keywords:** venous thromboembolism, cancer, blood disorders, pediatrics, thromboprophylaxis

## Abstract

Venous thromboembolism (VTE) in children is multifactorial and most often related to a combination of inherited and acquired thrombophilias. Children with cancer and blood disorders are often at risk for VTE due to disease-related factors such as inflammation and abnormal blood flow and treatment-related factors such as central venous catheters and surgery. We will review risk factors for VTE in children with leukemia, lymphoma, and solid tumors. We will also review risk factors for VTE in children with blood disorders with specific focus on sickle cell anemia and hemophilia. We will present the available evidence and clinical guidelines for prevention and treatment of VTE in these populations.

## Venous Thromboembolism in Children with Cancer

Venous thromboembolism (VTE) including deep venous thrombosis (DVT) and pulmonary embolism (PE) result in significant morbidity and mortality in individuals with cancer. VTE is a leading cause of death in adults with cancer (1). As care for critically ill children improves, the incidence of VTE in children is increasing (2). The general incidence of VTE in children ranges from 0.7 to 1.4 VTE/100,000 children and 53 VTE/100,000 hospital admissions (3–5). Children with cancer make up one of the largest subsets of patients who experience VTE (2). A study using the 1994–2009 Nationwide Inpatient Samples identified cancer as one of the primary risk factors for pediatric VTE-related hospital admissions (6). Other identified risk factors, central venous catheter (CVC) use, mechanical ventilation, and hospitalization of >5 days are common in this population.

VTE occurs in 2.1–16% of children with cancer (7–10). Rates vary based on the diagnostic imaging modality and whether VTE is symptomatic or asymptomatic. The rate is higher when patients who were screened for asymptomatic VTE are included (10, 11). Walker et al. conducted a population-based cohort study in the United Kingdom to compare rates of VTE between children with and without cancer. In this study, the absolute rate of VTE in children with cancer was 1.52 per 1,000 person-years (95% CI = 0.57–4.06) versus 0.06 per 1,000 person-years (95% CI = 0.02–0.15) in controls without cancer [hazard ratio of 28.3 (95% CI = 7.0–114.5)] (12).

The occurrence of VTE varies by cancer type (12). In a population-based cohort study utilizing national databases in the UK, the rate of VTE per 1,000 person-years in pediatric controls was 0.06 (95% CI 0.02–0.15) compared to 1.5 (95% CI 0.6–4.1) in all pediatric cancers, 0.9 (95% CI 0.1–6.1) in leukemia/lymphoma, 8.1 (95% CI 2.0–33.0) in soft tissue sarcoma/bone tumors, and 4.0 (95% CI 0.6–29.0) in other sites. In this report, there was no reported VTE in children with brain tumors. Much of the literature in children with cancer focuses on patients with acute lymphoblastic leukemia (ALL), the most common pediatric malignancy. A meta-analysis of children with leukemia reported VTE in 5.2% of children with ALL, but reported rates range from 1 to 36% (13–15). VTE occurs in 7–16% of patients with soft tissue sarcomas (16, 17). Interestingly, though thrombosis is often seen in adults with brain tumors, the incidence of thrombosis in children with brain tumors is quite low and ranges from <1 to 2.8% (7, 18, 19).

The etiology of VTE in children with cancer is multifactorial and includes genetic predisposition (thrombophilia), disease-related factors, and treatment-related factors including use of CVC, surgery, and chemotherapy. A Canadian multicenter case–control study of children with cancer identified age (≤2 and >10 years), blood group (non-O), and use of l-asparaginase as independent risk factors for DVT occurrence (20).

Cancer may be considered a hypercoagulable state. Albayrak et al. found activated coagulation and reduced fibrinolysis in children with ALL prior to chemotherapy (21). Giordano et al. identified thrombin generation at ALL diagnosis (22). The pathophysiology of this hypercoagulable state is related to secretion of cytokines and clotting factors by cancer cells (23). Pediatric tumors with mass effect impair blood flow and increase risk of VTE. In children with lymphoma, the presence of a mediastinal mass, which compresses upper extremity veins, increases the risk of thrombosis (24). Renal tumors with vascular invasion are also associated with VTE.

The majority of children with cancer have CVC placed for administration of chemotherapy and other supportive care. CVC is the most common risk factor for VTE in children with cancer. Reported rates of symptomatic catheter-related VTE range from 2.6 to 36.7%, and rates of asymptomatic catheter-related VTE range from 5.9 to 43% (25–27).

Certain aspects of cancer treatment increase the risk of thrombosis. Giordano et al. prospectively monitored changes in the coagulation parameters of children with ALL and showed that treatment for ALL altered the quantity and activity of numerous hemostatic proteins (22).

l-Asparaginase, which is used for treatment of ALL, is a well described risk factor for VTE. l-Asparaginase has widespread effects on coagulation including reduction in antithrombin. Steroids (particularly prednisone) increase factor VIII von Willebrand factor, which contributes to prothrombotic risk (23, 28, 29).

Additional prothrombotic risk factors include catheter-related blood stream and other infections in the immunocompromised patients as well as immobility during hospitalization, particularly in the post-operative period.

VTE results in serious consequences, including death. In a meta-analysis of children with ALL who developed VTE, about half of events occurred in the central nervous system (CNS). Fifteen to twenty percent of CNS thrombosis results in long-term neurologic sequelae (30–32). The Nordic Society of Paediatric Haematology and Oncology followed 20 patients with ALL and cerebral sinus venous thrombosis (CSVT), two of whom had deaths attributed to CSVT (33). Post-thrombotic syndrome (PTS), a chronic complication of VTE associated with chronic leg swelling, pain, and sometimes skin changes including ulceration, complicates both symptomatic and asymptomatic VTE (34). Polen et al. conducted a prospective cohort study of children with a history of cancer after CVC removal. PTS occurred in 30.5–34% of patients depending on the method of diagnosis. A history of CVC occlusion, DVT, or multiple CVC placements was associated with PTS (35).

### Treatment and Prevention of Venous Thromboembolism in Children with Cancer

There are no specific guidelines for treatment of VTE in children with cancer. The anticoagulants most commonly used in children are warfarin and heparins (36). There are ongoing clinical trials of anticoagulants in pediatric oncology patients, but no results have been published. Clinicians must consider the increased risk of bleeding in oncology patients who have thrombocytopenia and either withhold anticoagulation at low platelet counts or transfuse platelets at a lower threshold.

Thromboprophylaxis guidelines are well established for adults with cancer (37, 38). No such guidelines exist for children, even for those without cancer. There are limited data on prophylactic strategies of warfarin, low-molecular-weight heparin (LMWH), and antithrombin replacement in pediatric oncology patients with ALL (26, 39–46). None of these studies resulted in evidence-based strategies to prevent thrombosis. Nowak-Göttl et al. conducted an uncontrolled study of prophylaxis with LMWH in children with sarcomas; none developed VTE (47).

Several investigators have developed risk prediction models. Mitchell et al. published a validated, predictive model for the development of VTE in patients with ALL, which may be adapted to local patient population and practice to prevent VTE (43, 48). Bell et al. also published an approach to risk assessment and prophylaxis in this population (49). The Italian Association of Pediatric Hematology and Oncology made recommendations specifically for prolonged use of CVC in children with cancer and blood disorders. They recommend insertion of the CVC on the right side of the upper venous system and also the placing of the tip of the CVC at the right atrial–superior vena cava junction (50). These recommendations are based on studies showing higher rates of thrombosis when CVCs were placed on the left side and not placed at the right atrial–superior vena cava junction (51, 52).

### Summary

Children with cancer are at risk for developing VTE secondary to disease- and treatment-related factors and other inherited and acquired conditions. However, there is still much to be learned regarding risk factors, prevention, and treatment of VTE in this population.

## VTE in Children with Blood Disorders

VTE has been reported in children with acquired and inherited blood disorders. VTE occurs secondary to a combination of (1) underlying disease pathophysiology; (2) complications of disease; and (3) treatment of disease including medication, CVC placement, and surgery. See Table 1.

**Table 1:** **Risk factors for venous thromboembolism in children with cancer and blood disorders**.

	Cancer^a^	Sickle cell anemia^a^	Hemophilia
Central venous catheter	Chemotherapy	Chronic blood transfusions	Prophylaxis
	Supportive Care	Erythrocytapheresis	Immune tolerance induction
Surgery	Surgical management of cancer	Splenectomy^b^	Orthopedic procedures
		Cholecystectomy	
		Orthopedic procedures	
Immobility due to pain	Cancer and treatment-related pain	Vaso-occlusive pain crisis	Joint or muscle hemarthrosis
		Avascular necrosis	Chronic arthropathy
Infection	Sepsis and other invasive infection secondary to immunocompromised state	Sepsis secondary to functional and/or surgical asplenia	Sepsis
		Osteomyelitis	
		Acute chest syndrome	
Medication	Asparaginase	Erythropoietin	Factor replacement
	Corticosteroids		Bypassing agents
	Erythropoietin		

*^a^Underlying disorder is associated with hypercoaguability*.

*^b^Postsplenectomy state is associated with increased risk of VTE*.

A number of blood disorders are associated with inherent hypercoagulable states including [sickle cell anemia (SCA), see below]; vascular malformations (disseminated intravascular coagulation); hemophagocytic lymphohistiocytosis (hyper-inflammatory state); immune thrombocytopenic purpura (ITP); and autoimmune hemolytic anemia (AIHA) (53). Management of blood disorders with splenectomy is noteworthy, because the postsplenectomy state is associated with increased risk for VTE (54, 55). Splenectomy is indicated in children with SCA who suffer recurrent splenic sequestration and hypersplenism. Children with thalassemia may undergo splenectomy to increase red cell survival and decrease transfusion requirements. Splenectomy is an alternative to medical management in some cases of ITP, AIHA, and hereditary spherocytosis.

The rest of this section will focus on VTE in SCA and hemophilia.

### VTE in SCA

Sickle cell anemia is a hemoglobinopathy characterized by the presence of hemoglobin S. Clinical manifestations result from red cell hemolysis and vaso-occlusion. Acute complications include pain, acute chest syndrome (ACS), stroke, priapism, and splenic sequestration. Chronic complications include pulmonary hypertension, splenic dysfunction, and avascular necrosis (AVN).

Data are sparse in regards to the rates of VTE in children with SCA. Primary data include case reports of VTE (56–59), case series of catheter-related thrombosis (see below), and data within larger adolescent and adult cohort studies. In the Cooperative Study of Sickle Cell Disease, including 1,523 patients aged ≥15 years, the rate of first VTE was 5.2 per 1,000 person-years; including a PE rate of 3.6 per 1,000 person-years and isolated DVT rate of 1.6 per 1,000 person-years. Then, 11.3% had a least one VTE by age 40 years. Rates were highest in association with SS and Sβ^0^ thalassemia, and VTE was associated with higher risk of death.

Rates of VTE are expected to be higher than the general population, because, in addition to the baseline prothrombotic state, children and adolescents with SCA have other disease-related risk factors for VTE. Of note, VTE occurs starting at a younger age in adults with SCA compared to African-American controls; in a study of hospitalized patients with SCA, the mean age of patients with PE was 28 years compared to 57 years in controls and the mean age of patients with DVT was 31 years in patients with SCA compared to 54 years in controls (60).

The coagulation system is activated in SCA and SCA may be considered as a hypercoagulable state with higher levels of platelet activation, thrombin generation, and inflammation (61–66). Anticoagulants and antiplatelet agents are being studied as novel therapeutic agents for prevention complications in SCA (67).

The most common risk factor for VTE in children with SCA is presence of a CVC. CVC may be placed for short- and long-term venous access (68–71). A temporary CVC may be placed acutely during hospitalization for individuals with poor venous access, who are acutely ill and require intensive care, or in individuals with poor venous access requiring prolonged duration of intravenous therapy. Apheresis catheters are placed to facilitate exchange transfusion in the setting of stroke or ACS. Single or double lumen CVC is inserted to facilitate chronic red cell transfusions or exchange transfusion for primary and secondary stroke prevention (72). Jeng et al. reported a rate of catheter-related thrombosis of 0.99 per 1,000 catheter days in patients aged 1.4–30 years; 33% of the reported patients had catheter-related thrombosis (69). Shah et al. reported a rate of catheter-related thrombosis of 0.49 per 1,000 catheter days in patients aged 1–59 years; 41% of the reported patients had catheter-related thrombosis (68).

Other acquired prothrombotic risk factors in SCA include obesity, immobility, infection, and splenectomy. Although growth failure is a concern in children with SCA, rates of obesity, a known risk factor for VTE, are rising in children with SCA (73). Individuals with SCA may have chronic or acute immobility. Chronic immobility is related to chronic pain including pain from AVN. Acute immobility occurs during hospitalization for vaso-occlusive pain and other sickle cell-related complications and at the time of surgery for surgical procedures including abdominal (i.e., splenectomy, cholecystectomy) and orthopedic procedures (i.e., core decompression). Children and adolescents are at risk for invasive infections due to functional asplenia. Infectious complications include bacterial sepsis, ACS, and osteomyelitis. Other risk factors reported in adults with SCD SC and Sβ+ include higher hemoglobin and history or surgical splenectomy (74).

Clinicians must include VTE on the differential diagnosis of extremity and limb pain. If DVT occurs in the setting of vaso-occlusive crisis, diagnosis of DVT may be delayed if pain is attributed to vaso-occlusive crisis or other sickle cell-related complication. Chest pain occurring in hospitalized patients with SCA is most often attributed to vaso-occlusive crisis or ACS. PE must also be suspected in patients with significant chest pain and hypoxia. If PE is diagnosed, then extremity ultrasonography should be done to determine if thrombosis is truly embolic or *in situ*. Those with PE may be at higher risk for pulmonary hypertension (75).

d-Dimer is increased at baseline in SCA; therefore, d-dimer has lower prognostic significance in the diagnosis of VTE in SCA.

### Treatment and Prevention of VTE in Children with SCA

There are no specific guidelines for treatment of VTE in patients with SCA. Guidelines for prevention of VTE in hospitalized pediatric patients are still under development. Even in the adult population, there are no disease specific recommendations for SCA. Clinicians who take care of children and adolescents with SCA should evaluate patients for acquired prothrombotic risk factors and consider thromboprophylaxis if multiple risk factors are present.

### VTE in Children with Hemophilia

Although children with hemophilia are primarily at risk for bleeding and should be at lower risk of VTE due to clotting factor deficiency, thrombotic complications do occur. Thrombotic complications in this population are most often attributed to CVC, clotting factor replacement, and disease-related complications.

### Occurrence of VTE

Data are sparse on rates of thrombosis in children with hemophilia. Primary data include case reports and case series of children with catheter-related thrombosis.

As with SCA, CVC is the most common risk factor for VTE in children hemophilia. CVC is most often placed in children with hemophilia who require reliable venous access for prophylaxis, prophylactic clotting factor replacement administered one to four times per week, or immune tolerance induction, high-dose clotting factor administration up to 7 days per week for inhibitor eradication (76). Although Medeiros et al. reported a low rate of catheter-related thrombosis in children with hemophilia (77), subsequent publications document asymptomatic and symptomatic catheter-related thrombosis in patients with hemophilia (78–81). Risk may increase with duration of catheter presence (81). Even asymptomatic VTE are important to recognize given the risk and morbidity of PTS (79, 80). Consensus recommendations for use of CVC in hemophilia include the following: use of the smallest possible catheter diameter, position the catheter tip in the lower third of the superior vena cava, evaluate for catheter-related thrombosis after 2–4 years, and transition to peripheral access as soon as possible if thrombosis is detected (82). In general, due to the risk of catheter-related thrombosis, CVC should be avoided when possible and removed as soon as peripheral venous access is reliable for factor administration.

Other acquired prothrombotic risk factors include obesity, immobility, orthopedic surgery, infection, and high doses of clotting factor replacement. Rates of overweight and obesity are high in hemophilia (83, 84). This may be related to restricted activities. Children with hemophilia may suffer acute immobility due to joint and muscle bleeds and less commonly chronic immobility due to hemophilic arthropathy. VTE in persons with hemophilia ≤18 years has been described in the setting of major orthopedic surgery (85, 86). Orthopedic procedures are less common in children with hemophilia than in adults. Nonetheless, if a pediatric patient with hemophilia undergoes a major orthopedic procedure then patient should be fully assessed for any additional risk factors for thrombosis such as obesity and preventive measures may be considered (87, 88). Children with hemophilia are not inherently immunocompromised but may have CVC-related infection or infection in the setting of immunomodulatory therapy for inhibitors.

Patients with hemophilia and inhibitors often require high and frequent doses of bypassing agents for treatment of bleeding and factor replacement for ITI. Silvey et al. described cases of PE in young children with hemophilia A and high-titer inhibitors (89). Girolami et al. also described a higher frequency of thrombosis in inhibitor patients (90).

The most common etiologies of swelling in the lower or upper extremities in children with hemophilia are hemarthrosis, intramuscular bleed, and soft tissue bleeds. Therefore, clinicians likely have a lower index of suspicion for VTE as a cause of swelling and pain in the extremities, and the diagnosis of VTE in this patient population may be missed or delayed. Despite the bleeding phenotype of hemophilia, clinicians should maintain an index of suspicion for VTE in children with inherited bleeding disorders who have multiple prothrombotic risk factors.

### Treatment and Prevention of VTE in Children with Hemophilia

Martin and Key recently published an approach to treating patients with inherited bleeding disorders who need anticoagulant therapy (91). The authors point out that there are no standardized guidelines. When deciding whether or not to initiate anticoagulation, the patient’s bleeding phenotype must be balanced against the risk of developing or not treating thrombosis. In some cases, prophylactic clotting factor to increase factor levels >30% and decrease risk of bleeding may be required to allow for safe anticoagulation. Short-acting and reversible therapeutic agents are favored due to higher risk of bleeding. The intensity and duration of therapy must be carefully considered to minimize bleeding outcomes while achieving desired anticoagulant outcome.

### Summary

Children with blood disorders are at risk for VTE secondary due to disease-related factors, disease complications, and disease management. Care should be taken to target modifiable risk factors, to educate patients about signs and symptoms of VTE, and to consider thromboprophylaxis in the setting of multiple prothrombotic risk factors.

## Author Contributions

RK and CT designed and wrote the manuscript.

## Conflict of Interest Statement

The authors declare that the research was conducted in the absence of any commercial or financial relationships that could be construed as a potential conflict of interest.
